# Higher Coffee Consumption Is Associated With Slower Cognitive Decline and Less Cerebral Aβ-Amyloid Accumulation Over 126 Months: Data From the Australian Imaging, Biomarkers, and Lifestyle Study

**DOI:** 10.3389/fnagi.2021.744872

**Published:** 2021-11-19

**Authors:** Samantha L. Gardener, Stephanie R. Rainey-Smith, Victor L. Villemagne, Jurgen Fripp, Vincent Doré, Pierrick Bourgeat, Kevin Taddei, Christopher Fowler, Colin L. Masters, Paul Maruff, Christopher C. Rowe, David Ames, Ralph N. Martins, the AIBL Investigators

**Affiliations:** ^1^Centre of Excellence for Alzheimer’s Disease Research and Care, School of Medical and Health Sciences, Edith Cowan University, Joondalup, WA, Australia; ^2^Australian Alzheimer’s Research Foundation, Sarich Neuroscience Research Institute, Perth, WA, Australia; ^3^Centre for Healthy Ageing, Health Futures Institute, Murdoch University, Murdoch, WA, Australia; ^4^School of Psychological Science, University of Western Australia, Perth, WA, Australia; ^5^Department of Psychiatry, University of Pittsburgh, Pittsburgh, PA, United States; ^6^CSIRO Health and Biosecurity/Australian e-Health Research Centre, Herston, QLD, Australia; ^7^Department of Molecular Imaging and Therapy, Centre for PET, Austin Health, Heidelberg, VIC, Australia; ^8^The Florey Institute, The University of Melbourne, Parkville, VIC, Australia; ^9^Cogstate Ltd., Melbourne, VIC, Australia; ^10^National Ageing Research Institute, Parkville, VIC, Australia; ^11^Academic Unit for Psychiatry of Old Age, University of Melbourne, Melbourne, VIC, Australia; ^12^Department of Biomedical Sciences, Macquarie University, Sydney, NSW, Australia

**Keywords:** coffee, Alzheimer’s disease, AIBL, Australian Imaging Biomarkers and Lifestyle flagship study of ageing, caffeine, cognitive decline, Aβ-amyloid, dementia

## Abstract

**Background:** Worldwide, coffee is one of the most popular beverages consumed. Several studies have suggested a protective role of coffee, including reduced risk of Alzheimer’s disease (AD). However, there is limited longitudinal data from cohorts of older adults reporting associations of coffee intake with cognitive decline, in distinct domains, and investigating the neuropathological mechanisms underpinning any such associations.

**Methods:** The aim of the current study was to investigate the relationship between self-reported habitual coffee intake, and cognitive decline assessed using a comprehensive neuropsychological battery in 227 cognitively normal older adults from the Australian Imaging, Biomarkers, and Lifestyle (AIBL) study, over 126 months. In a subset of individuals, we also investigated the relationship between habitual coffee intake and cerebral Aβ-amyloid accumulation (*n* = 60) and brain volumes (*n* = 51) over 126 months.

**Results:** Higher baseline coffee consumption was associated with slower cognitive decline in executive function, attention, and the AIBL Preclinical AD Cognitive Composite (PACC; shown reliably to measure the first signs of cognitive decline in at-risk cognitively normal populations), and lower likelihood of transitioning to mild cognitive impairment or AD status, over 126 months. Higher baseline coffee consumption was also associated with slower Aβ-amyloid accumulation over 126 months, and lower risk of progressing to “moderate,” “high,” or “very high” Aβ-amyloid burden status over the same time-period. There were no associations between coffee intake and atrophy in total gray matter, white matter, or hippocampal volume.

**Discussion:** Our results further support the hypothesis that coffee intake may be a protective factor against AD, with increased coffee consumption potentially reducing cognitive decline by slowing cerebral Aβ-amyloid accumulation, and thus attenuating the associated neurotoxicity from Aβ-amyloid-mediated oxidative stress and inflammatory processes. Further investigation is required to evaluate whether coffee intake could be incorporated as a modifiable lifestyle factor aimed at delaying AD onset.

## Introduction

Worldwide, a high proportion of adults drink coffee daily, making it one of the most popular beverages globally. Coffee contains a range of bioactive compounds, including caffeine, chlorogenic acid, polyphenols and small amounts of vitamins and minerals (Spiller, 1984). Epidemiological studies suggest coffee has beneficial effects on various conditions including stroke (Larsson and Orsini, 2011), heart failure (Mostofsky et al., 2012), cancers (Wang et al., 2016), diabetes (Akash et al., 2014), and Parkinson’s disease (Ross et al., 2000). Alzheimer’s disease (AD) is a neurodegenerative disease characterized by progressive impairment of learning, memory and other cognitive deficits, with extracellular deposition of Aβ-amyloid (Aβ) protein within the brain leading to neuroinflammation, synaptic loss and neuronal death (Villemagne et al., 2013). Several studies suggest a protective role of coffee, with reduced risk of mild cognitive impairment (MCI) and AD reported (van Gelder et al., 2007; Eskelinen et al., 2009; Arab et al., 2011; Liu et al., 2016; Wierzejska, 2017; Wu et al., 2017). However, there are limited longitudinal data from cohorts of cognitively normal older adults describing associations of coffee consumption with distinct domains of cognition, and concurrently investigating potential neuropathological mechanisms underpinning any such associations.

Results from a meta-analysis conducted in 2016, which included nine published prospective cohort studies, found drinking one to two cups of coffee daily was associated with lower incidence of cognitive disorders (i.e., cognitive decline, cognitive impairment, AD, and all cause dementia) compared with less than one cup; studies ranged in follow-up from 1.3 to 28 years (Liu et al., 2016). Moreover, a recent cross-sectional analysis found self-reported lifetime intake of two or more cups of coffee per day was associated with lower rates of “Aβ positivity” (presence of significant brain Aβ), compared to less than two cups per day, in 411 non-demented older adults. However, current coffee intake was not related to “Aβ positivity” in this cohort, and neither current, nor lifetime intake, was related to cerebral mean cortical thickness or white matter hyperintensity volume (Kim et al., 2019). A second study investigating the effect of self-reported coffee consumption on brain volume found both high coffee consumption (≥ 4 cups per day) *and* low coffee consumption (no coffee or < 1 cup per day) to be associated with larger hippocampal volume cross-sectionally (*p* for test for quadratic trend = 0.001). There were no associations with volumes of the neocortex and striatum (accumbens, putamen, globus pallidus) or total intracranial volume. The cohort was homogeneous in nature, with all participants young Caucasian females (aged 23.2 ± 2.7 years), right handed with university education, and no history of smoking or drug/alcohol abuse (Perlaki et al., 2011). Conversely, higher self-reported coffee consumption (>3 cups/day compared to 0–1 cup/day) has also been associated cross-sectionally with smaller hippocampal volume, in 2914 individuals aged 59.28 ± 7.2 years (*p* = 0.033) (Araujo et al., 2016). However, this association was only observed when body mass index, previous coronary heart disease, alcohol consumption, and smoking were included in the statistical model, along with age, sex, and educational attainment (Araujo et al., 2016). Well-designed longitudinal cohort studies are necessary to further understand the influence of coffee consumption on AD phenotype and rates of decline in different cognitive domains.

The aim of the current study was to investigate the relationship of self-reported habitual coffee intake to rates of decline in varying cognitive domains, assessed using a comprehensive neuropsychological battery, over 126 months in 227 older adults classified as cognitively normal at baseline. Furthermore, we aimed to investigate whether habitual coffee intake was associated with rates of cerebral Aβ-amyloid deposition, or brain volume atrophy over the same time-period, in subsets of 60 and 51 participants, respectively. These investigations were conducted using data from the well-characterized Australian Imaging, Biomarkers and Lifestyle study of ageing (AIBL) (Fowler et al., 2021).

## Materials and Methods

### Participants

This report describes data from 227 cognitively normal participants who were enrolled at the Perth site of the AIBL study (Fowler et al., 2021) and who completed the Commonwealth Scientific and Industrial Research Organisation (CSIRO) food frequency questionnaire (FFQ). The AIBL study is a longitudinal study of cognitively normal individuals, as well as those with MCI and AD who are being assessed for prospective research into aging and AD. MCI and AD participants were excluded from the current analysis as the CSIRO FFQ requires estimations of food intake over the previous year, and there is potential for misclassification due to limited accuracy in recall.

All AIBL volunteers were aged 60 years and above at baseline, and excluded if they had a history of non-AD dementia, schizophrenia, bipolar disorder, significant current depression, Parkinson’s disease, cancer (other than basal cell skin carcinoma) within the last 2 years, symptomatic stroke, insulin-dependent diabetes, uncontrolled diabetes mellitus or current regular alcohol use exceeding two standard drinks per day for women or four per day for men. Further details regarding recruitment, assessment, inclusion and exclusion criteria are described in Fowler et al. (2021). The AIBL study is approved by the institutional ethics committees of Hollywood Private Hospital, Edith Cowan University, St Vincent’s Health and Austin Health (Fowler et al., 2021). Written informed consent was obtained from each participant prior to undertaking any procedures related to the study.

### Food Frequency Questionnaire

The CSIRO FFQ quantifies intake of over 200 foods and beverages (Lassale et al., 2009). The online version of the CSIRO FFQ was administered at the participant’s residence, over the phone or at our research institution. Online delivery of the questionnaire ensures completion of all questions as participants cannot proceed without finishing each section. Participants were able to complete the questionnaire in multiple sessions to prevent non-completion due to fatigue. An individual’s habitual coffee intake was determined as grams per day from responses to the question “How often do you drink coffee” with response options of number of cups consumed and frequency of consumption, e.g., “2 times per week,” “1 time per day,” etc. No delineation between caffeinated and de-caffeinated coffee, or methods of preparation (latte, cappuccino, no milk, soy milk, etc.) was possible from the FFQ responses. The FFQ was completed a mean of 3.38 years ± 0.9 standard deviations after the baseline assessment.

### Cognitive Assessments

A comprehensive neuropsychological battery of well-validated measures was administered according to standard protocols (described previously; Fowler et al., 2021) at baseline and up to seven additional assessments, 18 months apart, for up to 126 months. The battery assessed six cognitive domains—episodic recall memory, recognition memory, executive function, language, attention and processing speed, and the AIBL Preclinical Alzheimer Cognitive Composite (AIBL PACC; Donohue et al., 2014). Composite scores were calculated for each of these cognitive domains by first converting raw scores for individual measures to overall sample-based Z scores, then averaging Z scores for the relevant measures to compute a single composite score for each individual for that domain. Neuropsychological tests were assigned to one of the six cognitive domains via consensus among neuropsychologists, psychologists and neurologists involved in the AIBL study. Supplementary Table 1 lists the neuropsychological tests used to construct the cognitive domain composite scores. A “change” in clinical classification variable was also computed by assigning each participant a “0” if they remained cognitively normal from baseline throughout their study participation, and a score of “1” if they converted to MCI or AD status.

### Positron Emission Tomography

A subset of 60 participants underwent Positron Emission Tomography (PET) brain scans to quantify cerebral Aβ-amyloid burden at baseline, and on up to seven additional occasions, 18 months apart, for up to 126 months. Imaging with PET was conducted using 11C-PiB, 18F-NAV4694, or 18F-flutemetamol at the Western Australia PET and Cyclotron Service, Sir Charles Gairdner Hospital, and Oceanic Medical Imaging, Perth. Methodology for each tracer has been previously described (Bourgeat et al., 2018). Briefly, PET images were analyzed using CapAIBL (Bourgeat et al., 2015) and Aβ-amyloid burden was expressed using the Centiloid (CL) scale (Bourgeat et al., 2018). The CL scale provides a single continuous scale across the different Aβ-amyloid imaging tracers, where a value of “0” represents the typical Aβ-amyloid burden in young controls, and “100” the typical Aβ-amyloid burden seen in mild AD patients (Klunk et al., 2015). In addition to the continuous CL scale, Aβ-amyloid burden was also expressed as a categorical variable, defined as follows: “negative” = < 15 CL, “uncertain” = 15 CL to 25 CL, “moderate” = 26 CL to 50 CL, “high” = 51 CL to 100 CL, and “very high” = > 100 CL.

### Magnetic Resonance Imaging

A subset of 51 participants underwent Magnetic Resonance Imaging (MRI) brain scans at baseline, and on up to seven additional occasions, 18 months apart, to quantify gray matter, white matter, and hippocampal volumes. Participants underwent a 3D T1-weighted magnetization-prepared rapid acquisition gradient-echo sequence using the following acquisition parameters: in-plane resolution 1 X 1 mm, slice thickness 1.2 mm, repetition time (TR)/echo time (TE)/inversion time (TI) = 2,300/2.98/900, flip angle 9°, and field of view (FOV) 240 X 256. Magnetization-prepared rapid acquisition gradient-echo images for all participants were segmented into white matter, gray matter, and cerebrospinal fluid using an implementation of the expectation maximization algorithm (Van Leemput et al., 1999). Hippocampal extraction was performed using a multi atlas approach based on the Harmonized Hippocampus Protocol (Boccardi et al., 2015). All measures were corrected for total intracranial volume.

### Statistical Analysis

All statistical analyses were performed using Statistical Package for the Social Sciences (SPSS Inc., Chicago, IL) version 25. A *p-*value of < 0.05 determined a significant result.

Means, standard deviations and percentages are provided for the whole cohort and the brain imaging subsets (Table 1). Normality of the data was tested using the Kolmogorov–Smirnov test statistic. Cognitive domains and MRI brain region volumes were natural log transformed as necessary to better approximate normality.

**TABLE 1:** Descriptive statistics for the cognitively normal cohort and subsets with brain imaging.

	**Whole cognitively normal sample *n* = 227**	**Subset with Aβ-PET imaging *n* = 60**	**Subset with MR imaging *n* = 51**
Age, y	69.71 ± 6.0	70.44 ± 5.6	69.92 ± 5.2
Sex, male; n (%)	92 (40.5)	28 (46.7)	22 (49.0)
Presence of *APOE* ε4 allele; n (%)	61 (26.9)	23 (39.0)	22 (43.1)
Years of education, ≤ 12 y; n (%)	113 (49.8)	30 (50.0)	24 (47.1)
Daily coffee intake, g	280.90 ± 323.0	236.01 ± 232.6	237.02 ± 237.2
Time from baseline assessment to FFQ completion, y	3.38 ± 0.9	2.49 ± 1.3	2.67 ± 1.2
Daily energy intake, KJ	2243.30 ± 677.0	2429.83 ± 817.0	2327.08 ± 795.2

*Unless otherwise described, data are presented as mean ± standard deviation of the mean.*

*APOE, Apolipoprotein E; FFQ, food frequency questionnaire; g, grams; KJ, kilojoules; MR, magnetic resonance; PET, positron emission tomography; y, years.*

A series of repeated measures linear mixed model (LMM) analyses (using maximum likelihood estimation and an unstructured covariance matrix) were conducted to examine the relationship between coffee intake and time with respect to change in cognitive function. Coffee intake, age, Apolipoprotein (*APOE*) ε4 allele carrier status (presence or absence of ε4 allele; the most common genetic risk factor for AD; genotype determined as previously described (Fowler et al., 2021)), sex, education level (≤ 12 years and > 12 years), energy intake, and time from baseline to FFQ completion were entered as main effects, participant as a random factor, and coffee intake^∗^time as an interaction term. Models were then re-run with the cognitive function dependent variable exchanged for individual MRI brain region volumes or continuous PET Centiloid; however, education level was excluded from these neuroimaging models. Scanner location was included as a confounder in the MRI volume LMMs due to multiple scanners being utilized for MRI.

Multinomial logistic regression models calculated odds ratios for the investigation of the likelihood of exhibiting change in clinical classification from cognitively normal to MCI or AD, with increasing coffee intake. Multinomial logistic regression models additionally calculated odds ratios for the investigation of the likelihood of exhibiting “moderate,” “high,” or “very high” brain Aβ burden compared to “negative” burden over time, with increasing coffee intake. These models included the same covariates as the LMM analyses excluding education level.

All participants irrespective of their number of cognitive assessments or brain scans were used in the LMMs to reduce parameter variation, however, only those with two or more assessments were used in the calculation of the time-related changes dependent on coffee consumption. The number of brain imaging scans and cognitive assessments completed at each timepoint is reported in Supplementary Table 2.

## Results

The current cohort comprised 227 participants (40.5% male) with an average age of 69.7 years at baseline. Almost 27% were carriers of the *APOE* ε4 allele (genotypes ε2/4, ε3/4, or ε4/4) and nearly 50% had 12 or less years of education. Of the 60 participants included in the PET imaging analysis, 46.7% were male with an average age of 70.44 years, and almost 40% were carriers of the *APOE* ε4 allele. Of the 51 participants included in the MRI analysis, all were also included in the PET imaging analysis (supplemented by an additional *n* = 9 who did not undergo MRI). Forty-nine percent were male with an average age of 69.92 years, and over 43% were *APOE* ε4 carriers (Table 1).

Habitual coffee intake was positively associated with the cognitive domains of executive function (*p* < 0.01), attention (*p* < 0.05), and the AIBL PACC (*p* < 0.01), such that higher coffee consumption was associated with slower cognitive decline in these domains over 126 months (Table 2). Within the executive function, attention and AIBL PACC models, the following covariates contributed to variance in the outcome: for executive function, *APOE* ε4 allele carriage (*F* = 4.421, *p* = 0.037); for attention, age, and education (*F* = 4.779, *p* = 0.030; *F* = 6.335, *p* = 0.013, respectively); for the AIBL PACC, sex, age, and education (*F* = 8.987, *p* = 0.003; *F* = 30.131, *p* < 0.001; *F* = 20.655, *p* < 0.001, respectively). Moreover, odds ratios showed that higher coffee consumption was associated with lower risk of converting from cognitively normal to MCI or AD status over 126 months (β = −0.004; *p* = 0.006).

**TABLE 2:** Results of linear mixed models examining the association between coffee intake, cognitive decline, brain Aβ-amyloid accumulation, and brain volume atrophy in the cognitively normal cohort over 126 months.

**Change in cognitive domain or brain imaging measure**	**Slope estimate**	**F Statistic**	***p-*value**
Episodic recall	1.042^c^	0.395	0.532
Recognition	1.004^c^	0.079	0.780
Executive function	1.009^c^	7.974	0.006**
Language	1.003^c^	0.432	0.513
Attention	0.974^c^	4.284	0.044*
AIBL PACC	1.039^c^	9.954	0.003**
Aβ-amyloid Burden^a^	4.328	5.978	0.017*
Cortical gray matter volume^a^^b^	−2.033	2.940	0.138
White matter volume^a^^b^	−2.476	0.002	0.964
Left hippocampal volume^a^^b^	0.996^c^	0.136	0.713
Right hippocampal volume^a^^b^	0.997^c^	0.243	0.624

*Model includes coffee intake, age, APOE ε4 allele carrier status, energy intake, education level, time from baseline to FFQ completion, and sex as main effects.*

Bold indicates statistical significance (*p < 0.05, **p < 0.01).

*AIBL, Australian Imaging, Biomarkers, and Lifestyle study; APOE, Apolipoprotein E; FFQ, food frequency questionnaire; PACC, Preclinical Alzheimer Cognitive Composite.*

*^a^Does not include education level in model.*

*^b^Scanner location was included as a confounder in model.*

*^c^Cognitive domains and hippocampal brain region volumes were natural log transformed, and estimates were back transformed.*

**FIGURE 1:**
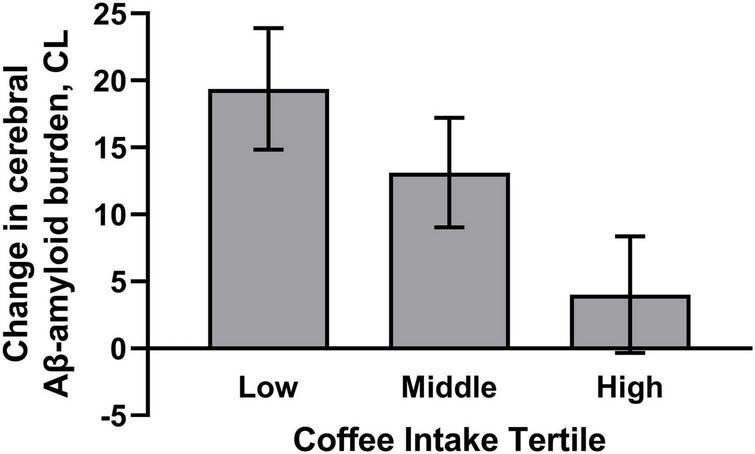
Plot demonstrates higher habitual coffee intake was associated with slower cerebral Aβ-amyloid accumulation. Graphical representation of mean change in cerebral Aβ-amyloid accumulation, ± SD, over 126 months, across tertiles of coffee intake (Low tertile = 0–26 g/day; Middle tertile = 36–250 g/day; High tertile = 360–750 g/day). Model includes age, apolipoprotein E (*APOE*) ε4 allele carrier status, sex, energy intake, and time from baseline to dietary questionnaire completion. Cerebral Aβ-amyloid burden was measured using positron emission tomography and expressed using the Centiloid (CL) scale.

Daily coffee intake was also associated with cerebral Aβ-amyloid burden, such that higher coffee consumption was associated with slower Aβ-amyloid accumulation over 126 months (*p* < 0.05; Table 2). Within the model, the following covariates contributed to variance in the outcome: energy intake, and time from baseline to FFQ completion (*F* = 4.860, *p* = 0.030; *F* = 4.865, *p* = 0.034, respectively). Furthermore, odds ratios showed that higher coffee consumption was associated with lower risk of transitioning to “moderate,” “high,” or “very high” cerebral Aβ-amyloid burden status over 126 months compared to not transitioning (β = −0.005, *p* = 0.035). Graphical representation of the change in cerebral Aβ-amyloid burden over 126 months, across coffee intake tertiles, demonstrates higher overall rates of Aβ-amyloid accumulation in those participants with “low” coffee intake (Figure 1). Of note, there were no significant differences in baseline Aβ-amyloid across the tertiles of coffee intake.

There were no associations observed between coffee intake and atrophy in gray matter, white matter, or hippocampal volume over 126 months.

## Discussion

The aim of this study was to investigate the relationship between habitual coffee intake and decline in multiple cognitive domains, over 126 months, in 227 cognitively normal individuals, accompanied by examination, in a subset of these individuals, of whether coffee intake was associated with rates of cerebral Aβ-amyloid accumulation, or brain volume atrophy.

Our results showed higher coffee consumption was associated with slower cognitive decline, specifically in the executive function and attention domains. Furthermore, higher consumption of coffee was also associated with slower decline in the AIBL PACC, which has previously been shown to reliably measure the first signs of cognitive decline in at-risk cognitively normal populations (Donohue et al., 2014). Consistent with these findings, higher coffee consumption was additionally associated with lower risk of transitioning from cognitively normal to MCI or AD status over the same time-period of 126 months.

In the neuroimaging subset, higher coffee intake was associated with slower cerebral Aβ-amyloid accumulation over 126 months, as well as lower risk of progressing to “moderate,” “high,” or “very high” Aβ-amyloid burden status over the same time-period. By contrast, coffee consumption was not associated with rates of brain volume atrophy.

The observed effect size estimates suggest that if the average cup of coffee made at home is 240 g, increasing intake from one to two cups per day could potentially provide up to 8% decrease in executive function decline over an 18-month period, and up to 5% decrease in cerebral Aβ-amyloid accumulation over the same time period. However, we readily acknowledge, that further longitudinal observational and intervention studies are required to substantiate this suggestion.

Whilst coffee consumption has previously been linked to decreased risk of MCI and AD (Solfrizzi et al., 2015; Wierzejska, 2017; Wu et al., 2017), until now, no prior studies have characterized the relationship of coffee intake to *rates* of decline in multiple cognitive domains. Nevertheless, consistent with the longitudinal findings of the current study, in a *cross-sectional* analysis of community-dwelling Dutch older adults (*n* = 2914; mean age 59.28 ± 7.2 years), consumption of > 3 cups of coffee per day was associated with better performance on an executive function measure (Araujo et al., 2016). However, the authors reported that this level of coffee consumption was also associated with worse performance on information processing speed and delayed memory measures, compared with 0–1 cups per day. The authors proposed an explanation of their findings whereby the neuro-stimulant effects of coffee on memory function are short-acting, and participants had not consumed coffee for at least 60 min preceding cognitive assessment (Araujo et al., 2016). In contrast to the current report, a study of Finnish twins found coffee consumption was not an independent predictor of cognitive performance in old age, among 2606 middle-aged twins assessed in 1975 and 1981, with a median follow-up of 28 years (Laitala et al., 2009). Cognitive status, however, was assessed using a brief telephone interview comprising a combination of a screen to identify potential dementia cases (TELE) and the Telephone Interview for Cognitive Status (TICS). Furthermore, coffee intake was assessed by the question “How many cups of coffee do you drink daily?” with respondents advised to answer “zero” if they did not drink coffee daily. In the present study, coffee intake over the previous year was used to generate a daily intake value on a continuous scale, such that drinking coffee less frequently than once a day would still yield an intake level; thereby generating a more realistic coffee consumption variable for analyses. Similar to the Finnish study, a study of 3494 Japanese males born between 1900 and 1919 and residing in Hawaii in 1964, reported that coffee intake was not associated with risk of cognitive impairment 25 years later. Like the Finnish study, cognition was not assessed using a comprehensive battery, instead a single screening test, the Cognitive Abilities Screening Instrument (CASI) was utilized, with impairment defined as scoring < 74 out of 100. Moreover, coffee intake was assessed via 24-h dietary recall, and the very specific cohort inclusion criteria limits the generalizability of results (Gelber et al., 2011).

Beyond the current study, only one other published report has examined the relationship between coffee intake and brain Aβ-amyloid (Kim et al., 2019). Consistent with the results of the current longitudinal study, where higher coffee intake was associated with slower cerebral Aβ-amyloid accumulation, this other study, comprising cross-sectional analysis of 411 non-demented older adults from South Korea, found lifetime intake of two or more cups of coffee per day was associated with lower rates of “Aβ positivity” (presence of significant brain Aβ-amyloid), compared to less than two cups per day. However, current coffee intake was not related to “Aβ positivity” in this South Korean cohort (Kim et al., 2019). MRI outcomes were also examined in this cross-sectional study, and consistent with our findings, neither current, nor lifetime coffee intake, was related to cerebral mean cortical thickness or white matter hyperintensity volume (Kim et al., 2019).

Precisely which constituents of coffee contribute to the positive outcomes described in the current study, and by others, remains to be determined. Yet, studies conducted in animal models of AD provide preliminary evidence to suggest the observed benefits are not due to caffeine alone. Both caffeine and crude caffeine (CC), a by-product of the coffee de-caffeination process, partially prevented memory impairment in AD mice, with CC producing a greater effect than pure caffeine. However, only CC consumption reduced the Aβ_1__–__4__2_ levels and the number of Aβ-amyloid plaques in the hippocampus of these animals. Nevertheless, *in vitro*, both caffeine and CC were shown to protect primary neurons from Aβ-induced cell death, and to suppress Aβ-induced caspase-3 activity (Chu et al., 2012). Multiple components of coffee, including cafestol and kahweol, have also been show to induce Nrf2 (Nuclear factor-erythroid factor 2-related factor 2) activation in *C. elegans* (Higgins et al., 2008). Nrf2-dependent upregulation of proteasomal activity has been directly implicated in protection against Aβ-mediated toxicity in cell culture models (Park et al., 2009); however, further work is needed to determine whether this is a related mechanism in humans. Moreover, dietary supplementation with Eicosanoyl-5-hydroxytryptamide (EHT), a minor component of coffee, for 6–12 months in rats, substantially ameliorated cognitive impairment, tau hyperphosphorylation, and elevated levels of cytoplasmic Aβ, potentially through its ability to increase phosphoprotein phosphatase 2A (PP2A) activity. These findings suggest EHT may also make a substantial contribution to the neuroprotective benefits associated with coffee consumption (Basurto-Islas et al., 2014).

The stimulant effect of coffee is attributed to the pharmacological activity of caffeine, acting as an antagonist of adenosine receptors in the brain (de Paulis et al., 2002). Adenosine regulates several physiological functions including sleep, cognitive performance, and memory, and its main role is to regulate neuronal excitatory synaptic transmission by inhibitory A_1_ receptors, and synaptic plasticity via facilitatory A_2_ receptors (Merighi et al., 2021). The effects of A_1_ and A_2_ receptor antagonists have been tested in animal models, where inactivation of both A_1_ and A_2_ receptors was shown to counteract age-related cognitive decline in rats (Prediger et al., 2005) and enhance release of several neurotransmitters including acetylcholine (Boison, 2006). Long-term caffeine administration to amyloid-beta precursor protein (APP) overexpressing mice has been shown to induce significant improvement in multiple cognitive tasks, as well as reduce levels of soluble Aβ-amyloid fragments compared to control mice (Arendash et al., 2006). The average daily intake of caffeine per mouse (1.5 mg) was the human equivalent of 500 mg caffeine, the amount typically found in five cups of coffee per day. Furthermore, caffeine reduced the production of Aβ_1__–__4__0_ and Aβ_1__–__4__2_ peptides in neuronal cell cultures from these same APP overexpressing mice (Arendash et al., 2006). Involvement of A_1_ receptors has also been observed in APP processing and tau phosphorylation *in vitro*, and the presence of A_1_ receptors in degenerating neurons with neurofibrillary tangles and Aβ-amyloid plaques in the hippocampus and frontal cortex suggest that these receptors may play a role in the pathogenesis of AD (Angulo et al., 2003). Whilst the current study cannot provide evidence that caffeine is causing the positive effects observed, the blocking of adenosine receptors by caffeine, leading to a decrease in Aβ-amyloid in the brain, and subsequent reduction of tau hyperphosphorylation is a potential mechanism that warrants further investigation.

There are some limitations that should be considered when interpreting our findings. Although we adjusted for known potential confounding factors there is a possibility of residual confounding due to other factors which were not measured. There is also the possibility of measurement error or recall bias with respect to the dietary data, which is especially true for questionnaire-based dietary exposures. However, coffee intake is less prone to misreporting due to its long-term habitual nature. Moreover, participants with cognitive impairment at baseline were excluded from the current analysis to reduce the likelihood of measurement error or recall bias. No data on mid-life coffee consumption was obtained from participants, consequently, potential deleterious or beneficial effects of coffee intake at midlife cannot be assessed in the current study. Finally, in our study it was not possible to differentiate between intakes of caffeinated or de-caffeinated coffee, nor to determine the potential consequences of varying methods of coffee preparation (e.g., brewing method, with or without milk or sugar etc.) on the associations observed. Nevertheless, strengths of our study include the long duration of follow-up, and comprehensive characterization of the cohort, including concurrent assessment of both multiple domains of cognition and neuroimaging biomarkers.

In summary, our results support the notion that habitual coffee intake may be a protective factor against AD. Specifically, increased coffee consumption could contribute to slower cognitive decline potentially by slowing the rate of cerebral Aβ-amyloid accumulation, and in doing so, ameliorate the associated neurotoxicity from Aβ-amyloid-mediated oxidative stress and inflammatory processes. Additional longitudinal observational and intervention studies are required to validate our findings and confirm this hypothesis. Such confirmation would corroborate the idea that coffee intake could be incorporated as a modifiable lifestyle factor aimed at delaying AD onset.

## Data Availability Statement

The datasets presented in this article are not readily available because the AIBL data are available only to authorized users. Requests to access the datasets should be directed to the following online form: https://ida.loni.usc.edu/collaboration/access/appApply.jsp?project=AIBL.

## Ethics Statement

The study involving human participants was reviewed and approved by the Ethics Commitees of Hollywood Private Hospital, Edith Cowan University, St Vincent’s Health and Austin Health. The patients/participants provided their written informed consent to participate in this study.

## Author Contributions

SG, SR-S, KT, CM, PM, CR, DA, and RM designed research. SG conducted research, primary responsibility for final content, and analysis for the manuscript. VV, JF, VD, and PB oversaw collection of and analyzed imaging data. SG and SR-S wrote the manuscript. VV, JF, VD, PB, KT, CF, CM, PM, CR, DA, and RM edited the final manuscript. All authors have read and approved the final manuscript.

## Conflict of Interest

VV has served as a consultant for IXICO. CM is an advisor to Prana Biotechnology Ltd., and a consultant to Eli Lilly. PM is a full-time employee of Cogstate Ltd. CR has served on scientific advisory boards for Bayer Pharma, Elan Corporation, GE Healthcare, and AstraZeneca, has received speaker honoraria from Bayer Pharma and GE Healthcare, and has received research support from Bayer Pharma, GE Healthcare, Piramal Lifesciences and Avid Radiopharmaceuticals. RM is founder of, and owns stock in, Alzhyme, and is a co-founder of the KaRa Institute of Neurological Diseases. The remaining authors declare that the research was conducted in the absence of any commercial or financial relationships that could be construed as a potential conflict of interest.

## Publisher’s Note

All claims expressed in this article are solely those of the authors and do not necessarily represent those of their affiliated organizations, or those of the publisher, the editors and the reviewers. Any product that may be evaluated in this article, or claim that may be made by its manufacturer, is not guaranteed or endorsed by the publisher.
