# Supplementary material for: Higher Coffee Consumption Is Associated With Slower Cognitive Decline and Less Cerebral Aβ-Amyloid Accumulation Over 126 Months: Data From the Australian Imaging, Biomarkers, and Lifestyle Study
Source: Front Aging Neurosci. 2021 Nov 19;13:744872. doi: 10.3389/fnagi.2021.744872 (PMC8641656; doi:10.3389/fnagi.2021.744872)
Supplement: Supplementary file 1 [file Table_1.DOCX]

**Supplementary Table 1: Cognitive domain composite score constituents.**

| **Cognitive domain composite** | **Tests** |
| --- | --- |
| Episodic recall memory | CVLT-II Long Delay Free Recall  Logical Memory 2  Rey Complex Figure Long Delay Free Recall |
| Recognition memory | CVLT-II ‘d prime’  Rey Complex Figure recognition total correct |
| Executive function | Letter Fluency  Category switching total correct |
| Language | BNT  Category Fluency |
| Attention and processing speed | Digit Symbol-Coding  Digit Span |
| AIBL PACC | MMSE  Digit Symbol-Coding  CVLT-II Long Delay Free Recall  Logical Memory 2 |

Abbreviations: AIBL PACC, Australian Imaging, Biomarkers and Lifestyle Study Preclinical Alzheimer Cognitive Composite; BNT, Boston Naming Test; CVLT-II, California Verbal Learning Test – Second edition; MMSE, Mini-Mental State Examination.

**Supplementary Table 2: Number of brain imaging scans and cognitive assessments completed at each timepoint.**

|  | **Baseline** | **18 Month Follow-Up** | **36 Month Follow-Up** | **54 Month Follow-Up** | **72 Month Follow-Up** | **90 Month Follow-Up** | **108 Month Follow-Up** | **126 Month Follow-Up** |
| --- | --- | --- | --- | --- | --- | --- | --- | --- |
| **Episodic recall** | 224 | 223 | 225 | 211 | 181 | 144 | 133 | 115 |
| **Recognition** | 223 | 224 | 225 | 211 | 181 | 143 | 133 | 115 |
| **Executive function** | 226 | 222 | 225 | 212 | 182 | 145 | 135 | 116 |
| **Language** | 154 | 222 | 225 | 212 | 182 | 145 | 133 | 116 |
| **Attention** | 225 | 223 | 225 | 211 | 181 | 144 | 134 | 115 |
| **AIBL PACC** | 225 | 222 | 225 | 210 | 181 | 144 | 134 | 115 |
| **PET** | 60 | 47 | 40 | 32 | 29 | 18 | 16 | 14 |
| **MRI** | 51 | 36 | 31 | 24 | 22 | 10 | 12 | 1 |

Abbreviations: AIBL, Australian Imaging, Biomarkers, and Lifestyle study; MRI, magnetic resonance imaging; PACC, Preclinical Alzheimer Cognitive Composite; PET, positron emission tomography.
